# Impact of Visceral Fat on Oxygenation Response to Prone Positioning in Mechanically Ventilated Patients With Severe COVID-19

**DOI:** 10.7759/cureus.110164

**Published:** 2026-06-03

**Authors:** Hiroki Matsui, Mariko Hanafusa, Tomoki Kawahara, Yuki Goto, Ayako Morita, Ukihide Tateishi, Koji Morishita, Takeo Fujiwara

**Affiliations:** 1 Department of Global Health Promotion, Institute of Science Tokyo, Tokyo, JPN; 2 Division of Cohort Research, Institute for Cancer Control, National Cancer Center Japan, Tokyo, JPN; 3 Department of Diagnostic Radiology and Nuclear Medicine, Institute of Science Tokyo, Tokyo, JPN; 4 Department of Trauma and Acute Care Surgery, Institute of Science Tokyo, Tokyo, JPN

**Keywords:** acute respiratory distress syndrom, covid-19, oxygenation, prone positioning, visceral fat

## Abstract

Background

Prone positioning therapy is internationally recommended as a cost-effective method to improve oxygenation and reduce mortality in patients with severe acute respiratory distress syndrome. This study investigated whether visceral fat (VF) area is associated with changes in oxygenation during prone positioning in mechanically ventilated patients with severe COVID-19 pneumonia. We further assessed whether VF modifies the magnitude of the oxygenation response to prone positioning.

Methods

We conducted a retrospective observational study of 89 critically ill COVID-19 patients who received mechanical ventilation and prone positioning therapy at a university hospital from April 2020 to November 2021. VF area was automatically quantified from admission chest CT, and patients were classified into high and low VF groups using a 100 cm² cutoff. The primary outcome was the ratio of the partial pressure of oxygen in arterial blood (PaO2) and fraction of inspiratory oxygen (FiO2) (P/F ratio) at four time points. Secondary outcomes included extracorporeal membrane oxygenation (ECMO) use. A multilevel analysis was performed to determine if VF area modified the therapy’s effectiveness, adjusting for covariates. An interaction term was included to assess whether the effect of prone positioning on oxygenation differed by VF level.

Results

Among 89 patients, baseline P/F ratios were similar between the low and high VF groups (147 vs 138). During prone positioning, oxygenation improved in both groups, and the low VF group showed a significantly higher P/F ratio than the high VF group (279 vs 228; p = 0.016). Interaction analysis showed that the difference in P/F ratios between groups was significantly greater during the prone positioning phase, suggesting that prone positioning amplified the effect of VF on oxygenation. This difference was no longer observed after returning to the supine position (181 vs 195). No significant differences were observed in ECMO use between the groups.

Conclusion

In severe COVID-19 pneumonia, the low VF group experienced significantly better oxygenation during prone positioning than the high VF group, with the difference being more pronounced during the prone phase. This difference was not observed after returning to the supine position. Prone positioning did not reduce the probability of ECMO initiation. Future studies should evaluate whether VF assessment can guide individualized prone positioning strategies and improve outcome-oriented patient selection.

## Introduction

Acute respiratory distress syndrome (ARDS) and prone positioning

Since the end of 2019, COVID-19 has infected over 776 million people and caused more than seven million deaths worldwide [1]. Because of the severity of COVID-19-associated pneumonia, it frequently results in ARDS, leading to severe hypoxemia and respiratory failure [2]. Prone positioning is a recognized intervention for improving respiratory status in ARDS, both prior to intubation and following initiation of mechanical ventilation [3]. In patients with ARDS, prone positioning is considered to reduce the inhomogeneous distribution of pleural pressure, increase lung volume, decrease atelectatic lung regions [4], and increase chest wall stiffness (i.e., reduce the ability of the chest wall to expand), as modulated by the abdomen, anterior chest wall, and posterior chest wall [5]. A landmark randomized controlled trial demonstrated a significant mortality benefit of prone positioning in patients with severe ARDS (ratio of partial pressure of oxygen in arterial blood (PaO2)/fraction of inspiratory oxygen concentration (FiO2) (P/F ratio) <150 mmHg) [6], whereas earlier trials failed to show survival benefit [7, 8]. On the basis of this evidence, prone positioning is currently recommended, though with some reservations, in both international and Japanese ARDS guidelines for patients with severe hypoxemia [9,10]. Recent advances have further emphasized the importance of individualized ventilation strategies that account for patient-specific factors such as lung morphology and body composition [11].

Prone positioning in COVID-19

COVID-19-associated ARDS shares pathophysiological features with classical ARDS but also exhibits distinct characteristics, including diffuse alveolar damage, a high prevalence of thromboembolic complications, and a particular pattern of lung morphology that may influence the response to positional therapy [2]. Prone positioning has been widely adopted for mechanically ventilated patients with severe COVID-19, and observational data suggest that it can meaningfully improve oxygenation in this population [12]. Studies have also demonstrated oxygenation benefits and reduced mortality with prone positioning in other complex ARDS populations, such as trauma patients [13]. However, prone therapy remains logistically demanding in intubated and sedated patients. Their inability to move independently and the presence of multiple tubes significantly increase the workload for healthcare providers, heightening the risk of complications. Some evidence suggests that extended prone sessions may alleviate these challenges by stabilizing lung mechanics and minimizing the frequency of required repositioning [14].

The role of body composition

One potential source of this heterogeneity is body composition. Visceral fat (VF) accumulation increases intra-abdominal pressure, restricts diaphragmatic excursion, and reduces total lung capacity, effects that may interfere with thoracic expansion and the redistribution of transpulmonary pressure achieved by prone positioning [15,16]. Body mass index (BMI), the most commonly used surrogate of obesity in clinical research, is a heterogeneous measure that does not capture fat distribution; individuals with identical BMI may differ substantially in visceral adiposity [17]. Observational studies have consistently shown that higher VF is associated with a greater risk of ICU admission, intubation, and death in COVID-19 [18-20]. Nevertheless, to our knowledge, no study has yet examined whether VF volume modifies the oxygenation response to prone positioning.

This study aims to investigate the relationship between a patient's VF volume and oxygenation levels throughout the course of prone positioning therapy, and to assess whether the oxygenation response differs by VF level at specific time points during and after the therapy.

## Materials and methods

This was a single-center retrospective observational study. Data were collected at Tokyo Medical and Dental University Hospital, a tertiary care emergency center and the main hospital for receiving COVID-19 patients in Tokyo, Japan. The research protocol was approved by Tokyo Medical and Dental University (Protocol No. M2023-157). As an observational study conducted using only existing clinical information, individual patient consent was not required.

Study population

The study included patients from April 1, 2020, to November 30, 2021, covering the first to fifth epidemic waves as defined by the Japanese Ministry of Health (n=665). The inclusion criteria were: Adult patients (aged ≥20 years) diagnosed with COVID-19 who developed severe pneumonia requiring ICU admission, received invasive mechanical ventilation during their ICU stay, and underwent at least one session of prone positioning therapy during their ICU stay. Patients under 20 years of age, patients in cardiopulmonary arrest at presentation, and patients with an ICU stay of less than 24 hours were excluded from the study. 

Patient outcomes were followed until hospital discharge or transfer to another medical facility; no post-discharge follow-up data were collected.

Prone position

Mechanical ventilation management and prone positioning therapy were implemented in alignment with established international guidelines, including the Surviving Sepsis Campaign: International Guidelines for Management of Sepsis and Septic Shock 2021 [21] and the ARDS clinical practice guidelines [9,10]. According to these guidelines, prone positioning was initiated in patients with a P/F ratio ≤200 and targeted to last for a minimum of 24 hours. Therapy duration was modified in response to changes in respiratory status, complication development, and staffing resources.

Oxygenation measurements

Improvement in oxygenation was selected as the primary outcome, given its potential to reduce the need for extracorporeal membrane oxygenation (ECMO) and its association with lower mortality [22]. Oxygenation was assessed using the P/F ratio. FiO2 values were extracted from electronic medical records and matched to respiratory settings recorded at the time of arterial blood gas sampling. Analysis was performed using the ABL800 FLEX blood gas analyzer (Radiometer, Copenhagen, Denmark), with results automatically transmitted to the ICU data system.

Oxygenation was assessed at four time points: before initiating prone positioning (t1), after initiating prone positioning (t2), before ending prone positioning (t3), and after returning to the supine position (t4). At each time point, the closest available blood gas measurement was used, as blood gas data were collected approximately every six hours as part of routine care rather than at fixed intervals relative to positional changes. Patients with incomplete blood gas data at any of the four designated time points were excluded from the final analysis.

ECMO initiation

ECMO initiation was assessed as a secondary outcome. A competing risks regression based on the Fine-Gray model was performed with ECMO initiation as the event of interest and in-hospital death as the competing event, adjusting for height, sex, age, and comorbidities. Patient transitions were tabulated as transfers, discharges, and deaths. Transfers included those who were still on ventilation but considered manageable in a secondary care setting and those who were transferred to best supportive care.

VF measurement

CT scan data were analyzed using the Ziostation2 Plus ZWS-2000 version 2.9.7.1 (Ziosoft, Inc., Tokyo, Japan) workstation, based on 5 mm thick images taken at or just before ICU admission. CT images at the level of the first lumbar vertebra were used for measurement, and this level was automatically identified by the Ziostation2 software. VF area was automatically quantified using a Hounsfield unit (HU) range of −160 to −70 as the tissue-specific threshold. Based on an established clinical threshold from prior research [23], VF was categorized into two groups: those with VF less than 100 cm² and those with VF equal to or greater than 100 cm².

Data collection

Patient demographic information such as age, weight, height, sex, smoking habit, and comorbidities (including hypertension and diabetes mellitus, as predefined clinical variables) was collected from electronic medical records as covariates. Treatment details, time elapsed since the start of prone positioning, complications, oxygenation, CT findings, and ECMO initiation were obtained from electronic medical records and the ICU data system. The Sequential Organ Failure Assessment (SOFA) score [24] and the Acute Physiology and Chronic Health Evaluation II (APACHE II) score [25] were calculated at the time of first ICU admission using data obtained from electronic medical records.

Statistical analysis

Continuous variables were presented as mean ± standard deviation (SD) or median (interquartile range (IQR)), while categorical variables were expressed as frequencies and percentages. All analyses were performed in Stata Statistical Software: Release 18 (StataCorp LLC., College Station, Texas, United States).

Mean P/F ratios at the four time points were plotted separately for the high and low VF groups to visually assess temporal changes in oxygenation. A multilevel regression analysis was performed to evaluate the impact of the VF group on the effectiveness of prone positioning, accounting for relevant covariates, using repeated P/F ratio measurements as the outcome. Patient ID was treated as a random effect. In Model 1, the analysis was adjusted for height, prone positioning status, time elapsed since the start of prone positioning, sex, and age. Model 2 included adjustments for comorbidities in addition to the variables in Model 1. In Model 3, adjustments for drug treatments were added to the variables in Model 2. An interaction term between the VF group and the time point was added to examine whether the oxygenation response to prone positioning was modified by VF level.

We analyzed ECMO initiation using a competing risks regression based on the Fine-Gray model, with in-hospital death as the competing event. Time was defined as the number of days from ICU admission to ECMO initiation, in-hospital death, or discharge/transfer, whichever came first. A competing risks regression was performed with ECMO initiation as the event of interest, adjusting for height, sex, age, and comorbidities.

## Results

**Figure 1:**
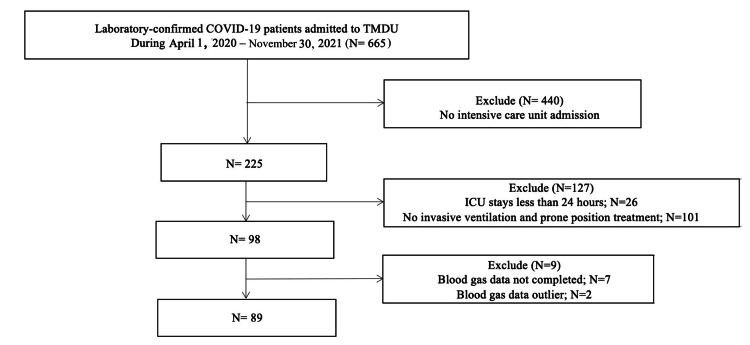
Participant flow chart TMDU: Tokyo Medical and Dental University Hospital

During the study period, 665 patients were admitted to the hospital, and 225 were admitted to the ICU. Excluding 101 patients who did not receive prone positioning therapy and 26 patients with ICU stays of less than 24 hours, a total of 98 patients received prone positioning therapy. After excluding cases with missing blood gas data and outliers with a P/F ratio exceeding 600, nine patients were further excluded, resulting in a final analysis of 89 patients (Figure 1).

There were 16 patients (18%) in the low VF group and 73 patients (82%) in the high VF group. The VF area in each group was 55 cm^2^ (SD, 29 cm^2^) and 199 cm^2^ (SD, 63 cm^2^), respectively. Overall, 74 patients (83%) were male, and the mean age was 62.6 years (SD, 13.5 years). There were no significant differences in SOFA scores, APACHE II scores, or baseline P/F ratios between the two groups.

The median duration of prone positioning therapy was 41 hours (IQR, 36-43 hours) in the low VF group and 41 hours (IQR, 24-43 hours) in the high VF group. The mean number of days from symptom onset to initiation of prone positioning therapy was 13.0 days (SD, 6.7 days) in the low VF group and 10.5 days (SD, 4.6 days) in the high VF group (Table 1). Adverse events were recorded in both groups. Pressure ulcers were the most frequent complication among adverse events, occurring in 60 cases (67%) overall, followed by catheter problems and accidental extubations, each occurring in two cases (2%). There was no significant difference in the incidence of complications between the two groups.

**Table 1:** Patients characteristics and outcome Continuous variables with an approximately normal distribution are shown as mean ± standard deviation (SD) and were compared with the independent-samples t-test; non-normally distributed continuous variables are shown as median (IQR) and were compared with the Wilcoxon rank-sum test. Categorical variables are presented as frequency (percentage) and were compared with Pearson’s χ² test. The “Test statistic” column lists the corresponding t values (for t-tests), z values from the Wilcoxon rank-sum test, or χ² values (for χ² tests). A p-value < 0.05 was considered statistically significant. BMI, body mass index; IHD, ischemic heart disease; CHF, congestive heart failure; IRRT, intermittent renal replacement therapy; SOFA, Sequential Organ Failure Assessment; APACHE, Acute Physiology and Chronic Health Evaluation; P/F, partial pressure of oxygen in arterial blood (PaO_2_)/fraction of inspiratory oxygen concentration (FiO_2_) ratio; ECMO, extracorporeal membrane oxygenation; IQR: interquartile range

Parameters		Total (N=89)	Low VF (<100) (n=16)	High VF (100+) (n=73)	Test static	p-value
Age (years), mean ± SD		62.6±13.5	67.2±15.0	61.7±13.1	t = 1.49	0.14
Sex, n (%)	Male	74 (83%)	8 (50%)	66 (90%)	χ^2^ = 15.29	<0.001
	Female	15 (17%)	8 (50%)	7 (10%)		
Height (cm), mean ± SD		168.5±8.4	163.8±8.7	169.6±8.0	t = 2.55	0.012
BMI, mean ± SD		26.2±4.9	22.4±2.8	27.0±4.8	t = -5.07	<0.001
Comorbidities, n (%)						
Smoking	Never	34 (38%)	7 (44%)	27 (37%)	χ^2^ = 6.92	0.031
	Past/current	43 (48%)	4 (25%)	39 (53%)		
	Missing	12 (13%)	5 (31%)	7 (10%)		
Hypertension		46 (52%)	6 (38%)	40 (55%)	χ^2^ = 1.57	0.21
Diabetes mellitus		33 (37%)	4 (25%)	29 (40%)	χ^2^ = 1.22	0.27
IHD or CHF		9 (10%)	2 (12%)	7 (10%)	χ^2^ = 0.12	0.73
Cerebral vascular disorder		6 (7%)	2 (12%)	4 (5%)	χ^2^ = 1.03	0.31
IRRT		4 (4%)	1 (6%)	3 (4%)	χ^2^ = 0.14	0.71
Radiological characteristics						
Visceral fat (cm2), mean ± SD		173±80	55±29	199±63	t = -8.90	<0.001
Lung opacity volume (%),mean ± SD		45.3±20.6	49.9±13.6	44.3±21.8	t = 0.98	0.33
Severity						
SOFA (1st ICU admission), median (IQR)		6 (4-8)	6 (4-10)	6 (3-7)	z = 0.80	0.43
APACHE II (1st ICU admission), median (IQR)		15 (10-21)	17.5 (12-24)	15 (10-20)	z = 1.26	0.21
Onset to prone (days), mean ± SD		11.0±5.1	13 .0±6.7	10.5±4.6	t = 1.78	0.078
P/F baseline, mean ± SD		139±50	147±52	138±50	t = 0.68	0.5
P/F following prone, mean ± SD		208±92	248±117	199±84	t = 1.92	0.058
P/F before returned to supine, mean ± SD		237±76	279±67	228±75	t = 2.45	0.016
P/F supine, mean ± SD		193±67	181±62	195±68	t = 0.78	0.44
Prone duration (hours), median (IQR)		41 (26-43)	41 (36-43)	41 (24-43)	z = -0.11	0.91
Adverse events, n (%)						
Pressure ulcer		60 (67%)	12 (75%)	48 (66%)	χ^2^ = 0.51	0.47
Catheter trouble		2 (2%)	0 (0%)	2 (3%)	χ^2^ = 0.45	0.5
Accidental extubation		2 (2%)	0 (0%)	2 (3%)	χ^2^ = 0.45	0.5
Treatment and clinical course						
Ventilator free days, median (IQR)		4 (1-9)	3 (0-5)	4 (1-11)	z = -1.22	0.22
Ventilator days, median (IQR)		13 (7-27)	10.5 (6.5-23.5)	13 (7-27)	z = -0.51	0.61
Outcome, n (%)	Discharge	14 (16%)	1 (6%)	13 (18%)	χ^2^ = 1.73	0.42
	Transfer	50 (56%)	11 (69%)	39 (53%)		
	All-cause in-hospital death	25 (28%)	4 (25%)	21 (29%)		
ECMO, n (%)		19 (21%)	2 (12%)	17 (23%)	χ^2^ = 0.91	0.34

Change in oxygenation

**Figure 2:**
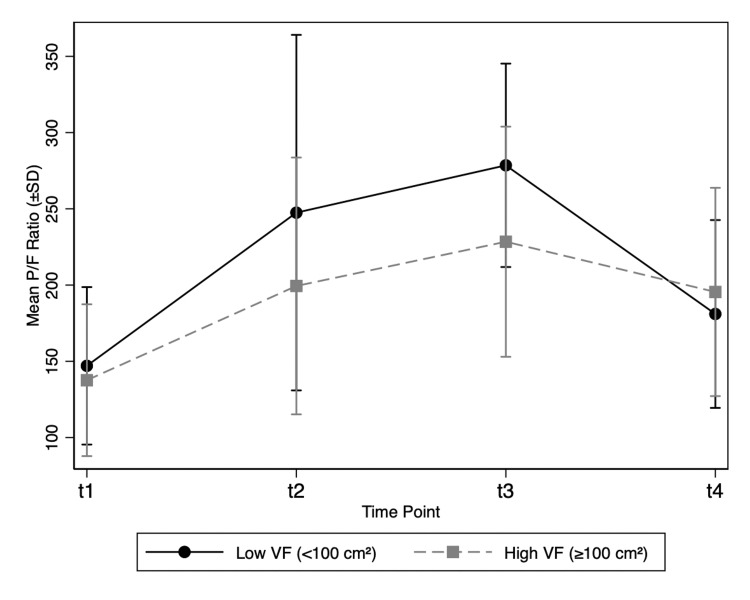
Changes in oxygenation at four time points during prone positioning therapy The dots represent the mean P/F ratio of each group, and the whiskers represent the standard deviation. t1: before prone positioning; t2: after initiation of prone positioning; t3: before return to the supine position; t4: after return to the supine position. P-values for between-group differences: t1, p=0.50; t2, p=0.058; t3, p=0.016; t4, p=0.44. VF: visceral fat; P/F: partial pressure of oxygen in arterial blood (PaO_2_)/fraction of inspiratory oxygen concentration (FiO_2_)

Mean P/F at each time point is shown in Table 1 and Figure 2. To test our hypothesis that VF volume modifies the oxygenation response to prone positioning, we compared P/F ratio changes at four time points between the low and high VF groups. The baseline mean P/F was 147 (SD, 52) in the low VF group and 138 (SD, 50) in the high VF group. The mean P/F after prone positioning was 248 (SD, 117) in the low VF group and 199 (SD, 84) in the high VF group. Both groups showed an improvement in oxygenation compared to baseline before returning to the supine position, with a particularly significant improvement observed in the low VF group (279 vs. 228, p=0.016). However, upon returning to the supine position, the P/F ratio decreased to 181 (SD, 62) in the low VF group and 195 (SD, 68) in the high VF group. No significant difference was observed after returning to the supine position (Figure 2).

Table 2 shows the results of a multilevel analysis to examine the association between the VF group and repeated P/F ratio measurements across time points. In model 3, we found that COVID-19 pneumonia patients with high VF were associated with a significantly smaller P/F ratio compared to those with low VF (coefficient, -31.5 (95% CI, -60.2 to -2.8)).

**Table 2:** Adjusted association of a prone positioning therapy with visceral fat, comorbidities, and pharmacological intervention HT, hypertension; DM, diabetes mellitus; IHD, ischemic heart disease; CHF, congestive heart failure; IRRT, intermittent renal replacement therapy

Parameters		Model 1			Model 2			Model 3		
		Coefficient	95%CI		Coefficient	95%CI		Coefficient	95%CI	
Visceral fat (cm2)	Low	ref.			ref.			ref.		
	High	-29.5	-58.8	-0.2	-32.1	-60.7	-3.6	-31.5	-60.2	-2.8
Height (cm)		1.3	-0.5	3.1	1.4	-0.4	3.1	1.4	-0.4	3.1
During prone		57.5	45.3	69.7	57.5	45.3	69.7	57.4	45.3	69.6
Time difference from prone start (hours)		1.2	0.9	1.6	1.2	0.9	1.5	1.2	0.9	1.6
Sex	male	ref.			ref.					
	female	2.6	-37.0	42.1	3.7	-34.0	41.5	-0.2	-38.2	37.8
Age (year)		-0.2	-1.0	0.7	0.02	-0.8	0.9	0.0	-0.8	0.9
Comorbidities	HT	--	-	-	15.2	-5.6	35.9	15.7	-5.0	36.4
	DM	-	-	-	-7.2	-27.8	13.3	-7.0	-27.7	13.7
	IHD CHF	-	-	-	-32.1	-67.1	2.9	-34.7	-69.8	0.4
	IRRT	-	-	-	-41.2	-89.9	7.5	-45.6	-95.3	4.1
Treatment	Remdesivir	-	-	-	-	-	-	-8.5	-29.9	12.9
	Corticosteroid	-	-	-	-	-	-	14.2	-7.3	35.8

ECMO initiation

ECMO initiation occurred in two cases (12%) in the low VF group and 17 cases (23%) in the high VF group. Using the Fine-Gray competing risks regression model with in-hospital death as the competing event, the subdistribution hazard ratio (SHR) for ECMO initiation in the high VF group compared to the low VF group was 1.51 (95% CI, 0.31-7.39), indicating no statistically significant difference between the two groups, as the confidence interval crossed 1.

## Discussion

The main findings of this study are that, in the multilevel analysis using repeated P/F measurements, patients with low VF consistently demonstrated higher oxygenation levels throughout the course of prone positioning therapy compared to those with high VF. Specifically, the interaction analysis revealed that the difference in P/F ratios between VF groups was significantly greater during the prone positioning phase; however, this difference was no longer significant after the prone position was discontinued. In addition, the probability of ECMO initiation did not differ significantly according to VF level. These findings suggest that VF may influence the short-term oxygenation response to prone positioning, whereas long-term oxygenation outcomes are likely determined by multiple factors beyond VF. There were no significant differences in baseline severity indicators, including SOFA scores, APACHE II scores, and baseline P/F ratios, between the two groups, suggesting that the observed differences in oxygenation response were unlikely to be explained solely by differences in baseline disease severity. Regarding clinical outcomes, the discharge rate was numerically higher in the high VF group compared to the low VF group (18% vs. 6%); however, this difference did not reach statistical significance (p=0.42). Given the small sample size, particularly in the low VF group (n=16), this observation should be interpreted with caution and warrants further investigation in larger cohorts.

According to several previous observational studies, an increase in VF volume is associated with a higher risk of severe COVID-19, leading to higher probabilities of ICU admission, intubation, and death [18-20]. BMI, frequently used as an indicator of obesity in research and clinical practice, is a heterogeneous measure. Even among individuals with the same BMI, there can be significant differences in fat distribution [17]. Therefore, VF volume is considered a more accurate metric for risk stratification than BMI [20]. To our knowledge, no previous studies have specifically examined the relationship between VF volume and oxygenation response to prone positioning in mechanically ventilated patients with severe COVID-19.

The prone position has been noted to temporarily improve oxygenation but not to enhance survival rates [8]. Subgroup analyses and subsequent studies have suggested that longer durations of the prone position may be more beneficial for patients with more severe hypoxemia. However, the factors influencing oxygenation improvement during the prone position have not been fully elucidated.

In this study, we hypothesized that VF could impact the inhomogeneous distribution of transpulmonary pressure, lung volume, and chest wall compliance, and thus examined its influence on oxygenation improvement during the prone position. The high VF group showed lower oxygenation levels during the prone positioning phase than the low VF group, with a statistically significant difference observed before returning to the supine position (t3, p=0.016). This may be because, in patients with high VF, prone positioning leads to greater chest wall restriction and increased intra-abdominal pressure, resulting in less improvement in oxygenation during the prone phase. However, factors such as postural drainage of secretions and recruitment of previously collapsed alveoli, which may have lasting effects, might have contributed to sustaining oxygenation improvement after returning to the supine position. The precise mechanisms by which these effects were more pronounced in the high VF group remain unclear and warrant further investigation.

Strengths

The strengths of this study include the enrollment of severely ill patients requiring mechanical ventilation or ECMO, and the consistent method used for measuring VF, which did not rely on the skill of radiologists. This contributed to data consistency and reproducibility, enhancing the reliability of the study. Regarding adverse events, pressure ulcers were the most frequent complication in both groups, occurring in 75% of the low VF group and 66% of the high VF group, with no significant difference between the groups. This finding is consistent with previously reported complication rates for prone positioning and suggests that VF level does not meaningfully affect the risk of prone positioning-related complications.

Limitations

Several limitations also need to be addressed. This research was conducted in a single institution in Japan; thus, generalizability needs further investigation in other settings. Although not all facilities might be equipped to easily measure VF, when taking a chest CT, the amount of VF can be visually interpreted. Additionally, follow-up data for transferred patients were not collected, as post-transfer follow-up was not feasible due to the retrospective nature of the study and the absence of a systematic follow-up protocol. Furthermore, the small sample size, particularly in the low VF group (n=16), may introduce bias in between-group comparisons. Finally, the absence of a control group limits causal inference.

## Conclusions

This study demonstrates that VF level modifies the short-term oxygenation response to prone positioning in mechanically ventilated patients with severe COVID-19, with patients in the low VF group showing greater oxygenation improvement during the prone positioning phase. However, this advantage was not sustained after returning to the supine position, and no significant difference in ECMO initiation was observed between groups. These findings suggest that VF may serve as a useful parameter for predicting the oxygenation response to prone positioning and highlight the potential value of incorporating body composition assessment into individualized ventilation strategies for patients with severe COVID-19.
